# Supplementary material for: Cloud BioLinux: pre-configured and on-demand bioinformatics computing for the genomics community
Source: BMC Bioinformatics. 2012 Mar 19;13:42. doi: 10.1186/1471-2105-13-42 (PMC3372431; doi:10.1186/1471-2105-13-42)
Supplement: Additional file 1 — Supplementary 1 Cloud BioLinux software documentation in the form of a mini, self-contained website. Users need to download and uncompress the .zip file, and open through a web browser the "index.html" file available on the main directory. (ZIP 1823 kb). [file 1471-2105-13-42-S1.ZIP › Cloud-BioLinux-Package-Documentation/docs/rdp_classifier.html]

Bio-Linux Software Documentation Pages

Back to search form

## rdp\_classifier

|  |  |
| --- | --- |
| Name | rdp\_classifier |
| Description | **rdp\_classifier**is a naive Bayesian classifier developed for rapid taxonomic placement based on rRNA sequence data. It provides taxonomic assignments from domain to genus, with confidence estimates for each assignment.  The version of rdp\_classifier released on Bio-Linux was trained on data from the Taxonomic Outline of Bacteria and Archaea.  You can see the command line options by typing:  `rdp_classifier` |
| Homepage | http://rdp.cme.msu.edu/ |
| Remote Documentation | http://rdp-classifier.sourceforge.net/      http://rdp.cme.msu.edu/ |
